# Quadratus Lumborum 2 Block as the Sole Anesthetic Technique for Open Hernia Repair in Multimorbid Patients

**DOI:** 10.7759/cureus.9697

**Published:** 2020-08-12

**Authors:** Julius Balogh, Angela Chen, Tejaswi Marri, Johanna B De Haan, Sara Guzman-Reyes

**Affiliations:** 1 Anesthesia and Critical Care, University of Texas at Houston, McGovern Medical School, Houston, USA; 2 Pediatric Anesthesiology, UT Houston, McGovern Medical School, Houston, USA; 3 Anesthesia, University of Texas at Houston, McGovern Medical School, Houston, USA; 4 Anesthesiology, McGovern Medical School, University of Texas Health Science Center, Houston, USA

**Keywords:** regional anesthesia, quadratus lumborum block, ultrasound-guided, ultrasound guided blocks and vascular access, abdomen ventral hernia, conduction anesthesia

## Abstract

Ultrasound-guided quadratus lumborum (QL, QL1-3) blocks have been used extensively for perioperative pain control for patients undergoing abdominal procedures. These blocks provide a more widespread and longer-lasting analgesic effect compared to the transversus abdominis plane (TAP) block. While QL blocks have been used as an adjunct in multimodal postoperative pain control, they are rarely used as the sole anesthetic technique for abdominal surgeries.

We report the cases of two high-risk multimorbid patients requiring urgent open umbilical hernia repairs secondary to incarceration or obstruction. Bilateral QL2 blocks were utilized as the sole anesthetic technique to reduce anesthetic risk, with positive outcomes.

Utilization of the QL2 block technique for our patients enabled avoidance of general anesthesia in these high-risk patients because of the extensive area of anesthesia they provide when compared with the TAP and QL1 block techniques. The advantages of the QL2 block for high-risk patients include immediate perioperative pain control, reduced use of muscle relaxants, reduced opioid analgesic requirement for postoperative pain management, and enhanced postoperative recovery. Disadvantages include potential for local anesthetic toxicity, neural injury, or failure of the block. While regional anesthetic techniques may be beneficial options for those patients who are not candidates for general anesthesia, more studies in which these techniques are used need to be performed to determine the widespread efficacy and adequacy of this method.

## Introduction

Hernia (incisional, ventral ,inguinal, Spigelian, obturator, lumber, and femoral) repairs are generally routine surgeries, typically performed under general anesthesia, and most often in an outpatient setting [1]. Patients with multiple medical comorbidities may not be candidates for general anesthesia. In addition, incarceration of either the small or large intestine can create an obstruction requiring emergent repair with very little time to optimize the patient. Regional anesthesia is considered a suitable option for these patients, as it can provide effective analgesia and has been associated with positive perioperative outcomes [2]. Ultrasound-guided quadratus lumborum (QL) blocks, first described by Blanco have been used for postoperative pain following abdominal surgeries and as the primary anesthetic technique for repair of inguinal hernias [3-6]. There is little literature describing the use and efficacy of this technique for surgical anesthesia for the repair of umbilical hernias. The QL2 block (deposition of local anesthetic at the postero-lateral aspect of the QL) provides sensory analgesia for dermatomal levels T4-L1, which are the primary dermatomes innervating the abdominal wall and groin [7-8].

We describe a case series of two patients with multiple medical comorbidities who required open hernia repairs but were inappropriate candidates for general or neuraxial anesthesia. Both patients received bilateral surgical QL2 blocks with light sedation for their surgical procedures and experienced no postoperative complications.

## Case presentation

Case 1 

**Figure 1:**
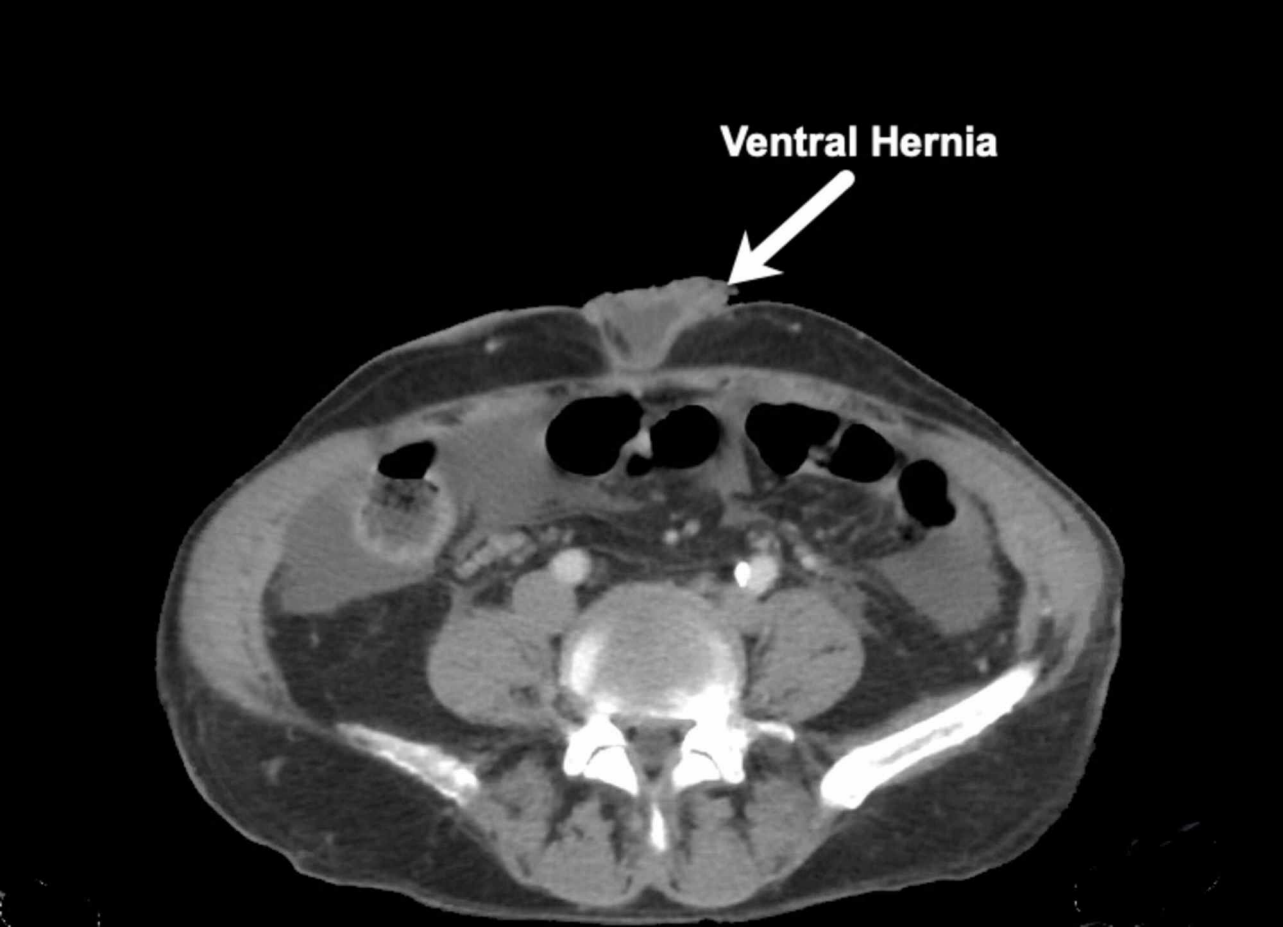
Case 1: Coronal CT scan.

**Figure 2:**
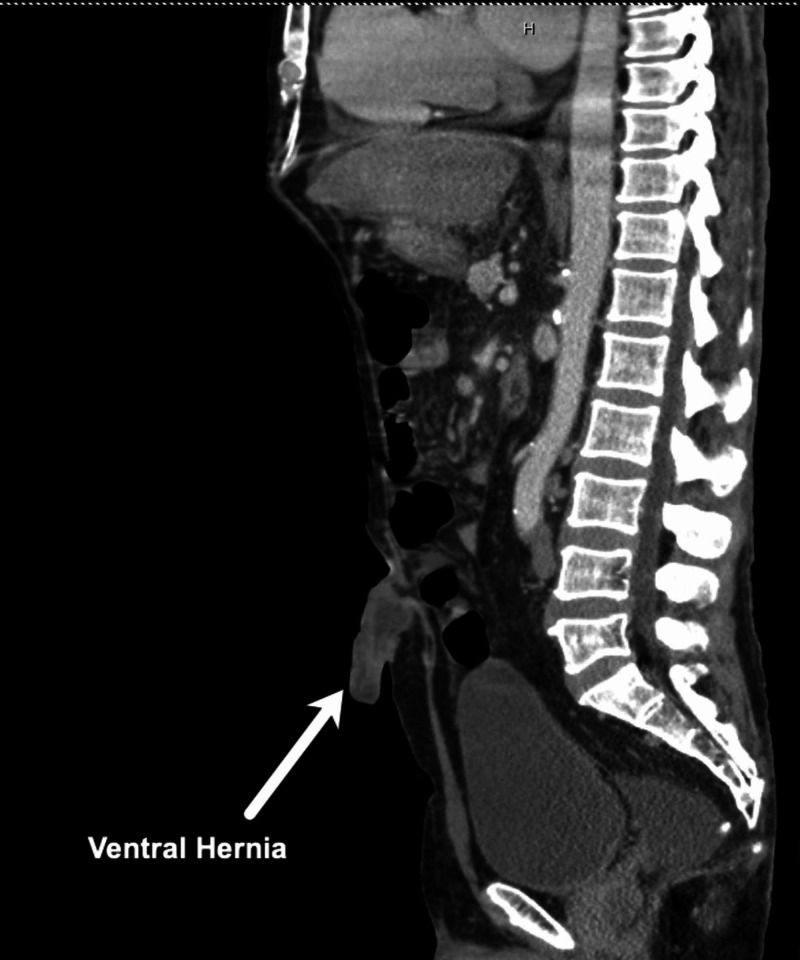
Case 1: Sagittal CT scan.

A male in his early sixties presented to the ED with an umbilical hernia (Figures 1-2).

He was leaking ascitic fluid from the ulcerated hernia sac. His medical history included congestive heart failure (CHF) secondary to cardiomyopathy with an ejection fraction (EF) of less than 10%, coronary artery disease (CAD), end stage liver disease (ESLD), and chronic kidney disease (CKD). Our general surgery colleagues recommended that he undergo an open umbilical hernia repair. However, it was determined that the patient would require extensive preoperative medical optimization, re-evaluation of cardiac function, and hemodynamic stabilization prior to surgical intervention. The cardiologist deemed the patient an above average risk for cardiac complications with surgery (cardiac complications 0.5% [average = 0.1%], death 1.0% [average = 0.0%]). The gastroenterologist determined that the patient was a high risk for intrabdominal surgery with a mortality rate of 20%-25% secondary to his Na-MELD score of 21 and Child-Pugh Score B. 

**Figure 3:**
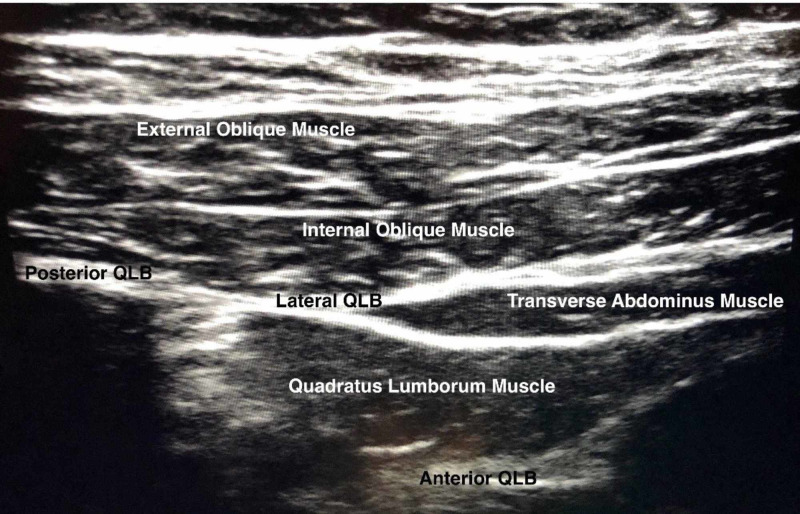
Sonoanatomy of QLBs. QLBs, quadratus lumborum blocks

The patient’s symptoms progressed and he was evaluated by an anesthesiologist and deemed to be high risk for both general and spinal anesthesia (GA) at that time and, after multi-disciplinary discussion, surgical regional block combined with monitored anesthesia care (MAC) was deemed the most appropriate management [9]. Informed consent was obtained from the patient for these procedures as well as research. Bilateral QL2 single shot blocks were placed in the preoperative area with adherence to standard American Society of Anesthesiologists (ASA) monitoring and 1 mg IV midazolam was administered. A high frequency linear ultrasound probe was placed in the axial plane in the midaxillary line to visualize the expected three abdominal layers (transversus abdominis, external oblique, and internal oblique) (Figure 3).

**Figure 4:**
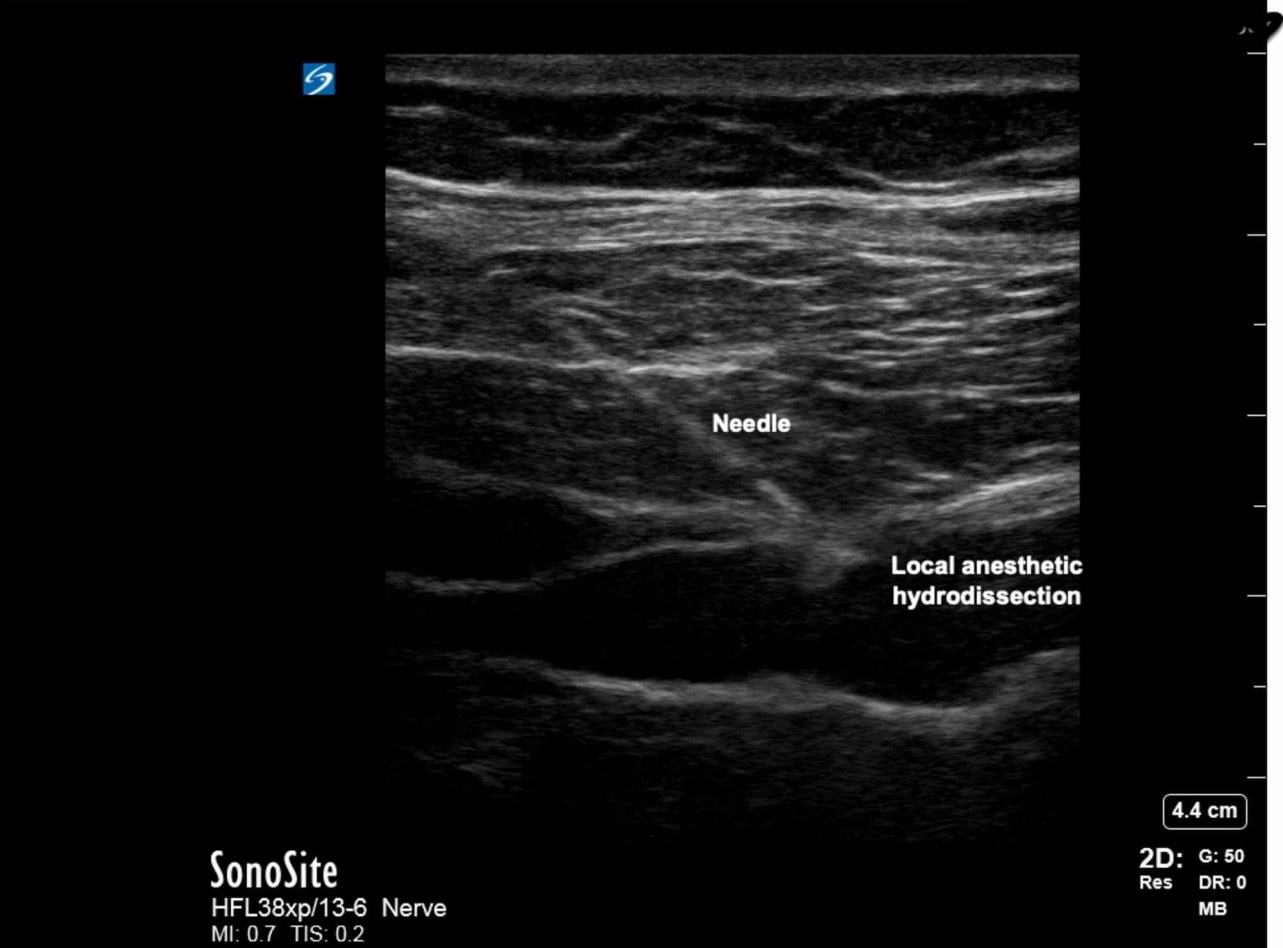
Ultrasound image of local anesthetic hydro-dissection for QL 2 block (QL2; posterior QLB). QL, quadratus lumborum; QLB, quadratus lumborum block

The probe was moved posteriorly until the QL was confirmed. The posterior aspect of the QL was identified. The skin was prepped using standard aseptic technique, and the needle inserted and advanced to the posterior aspect of the QL muscle. Proper positioning of the needle tip was confirmed by hydrodissection , and 20 mL of 0.5% ropivacaine was injected into the lateral interfascial triangle (LIFT) behind the QL muscle (Figure 4) [10].

Satisfactory expansion of the intermuscular plane due to spread of the local anesthetic was observed. This was repeated on the opposite side. Surgery proceeded 40 minutes after the blocks were performed. Anesthesia was tested across the necessary dermatomal levels prior to surgical sterile prep. Intraoperative sedation was achieved with a 0.1 mcg/kg/min infusion of remifentanil. During the procedure, the patient maintained a patent native airway and vital signs remained at baseline. Following completion of surgery the patient was transported to the post anesthesia care unit (PACU) awake and oriented, with a visual analog pain score (VAS) of 0/10. His postoperative pain score on the floor was 0/10 at rest and 2/10 with movement. The pain score was the highest 12 hours post operatively at 5/10. The patient was discharged on post-operative day 1, with well-controlled pain, and he was voiding and ambulating without difficulty. He received multi-modal pain control on discharge (acetaminophen, gabapentin, and tramadol). He was followed by surgery in their clinic and he is reported to be doing well without any complication. 

Case 2

**Figure 5:**
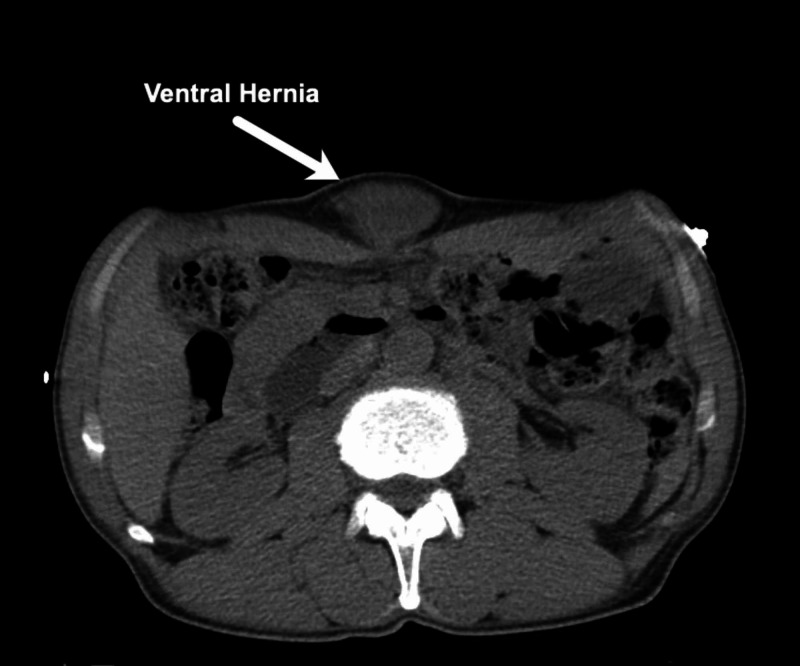
Case 2: Coronal CT scan.

**Figure 6:**
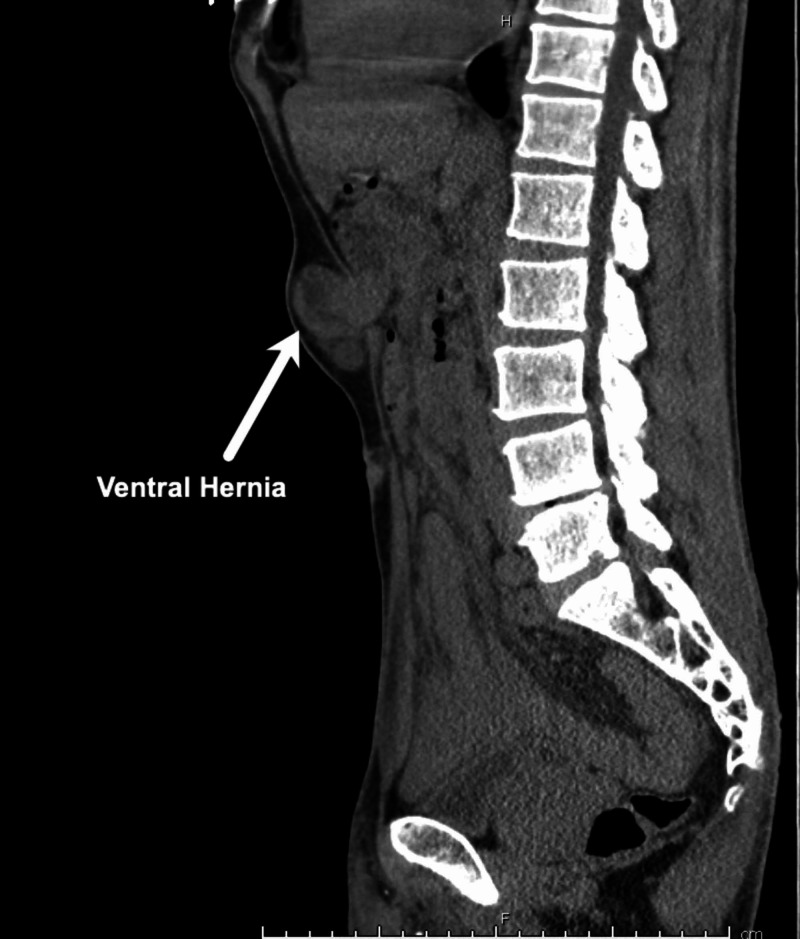
Case 2: Sagittal CT scan.

A male in his late fifties presented to the ED with significant nausea and vomiting and on examination was found to have an incarcerated ventral hernia (Figures 5-6).

He was hyponatremic (131 mmol/L), hypokalemic (3.2 mmol/L), hypochloremic, and hypocalcemic (ionized: 0.88 mmol/L). Past medical history included CHF secondary to nonischemic cardiomyopathy, an EF < 10%, CAD, chronic obstructive pulmonary disease (COPD), ESRD, hypertension (HTN), and a history of cocaine abuse. He was admitted to the ICU for optimization of his medical conditions and the decision made to proceed with emergent hernia repair secondary to small bowel obstruction. 

The patient was not a candidate for general anesthesia due to his significant comorbidities. Our anesthesia team evaluated the patient and proposed regional nerve blocks and MAC for the surgery. In the preoperative area, standard ASA monitors were applied, and the patient received bilateral QL2 single shot blocks. Block technique was identical to that described above. Surgery proceeded 45 minutes after the blocks were performed. Anesthesia was tested across the necessary dermatomal levels prior to surgical sterile prep and was deemed to be adequate. Intraoperative sedation was achieved with a 0.1 mcg/kg/min infusion of remifentanil and dexmedetomidine 0.04 mcg/kg/h. Additional medication was administered to reduce sympathetic stimulation as well as to provide sedation due to the patient’s nervousness. During the procedure the patient maintained a patent native airway and vital signs remained at base line. Following completion of the operation the patient was transported to PACU awake and oriented, with a pain score 0/10. He was discharged on postoperative day 11 after optimization of his medical conditions. Upon discharge, his pain was well controlled, he was voiding and ambulating without difficulty, and his symptoms of nausea and vomiting had resolved. He reported no pain and was doing well overall at his four-week post-operative follow up appointment in surgery clinic. 

## Discussion

Historically, regional anesthesia has been proposed as a valid alternative to GA in patients with multiple comorbidities. Much of the literature details surgical blocks for extremity surgeries, or neuraxial techniques for cesarean section deliveries or urologic procedures. There is a paucity of literature on the use of QL blocks as the primary surgical anesthetic. Given the significant cardiovascular risk factors as well as the multiple comorbid conditions of the patients described herein, we elected to perform surgical posterior QL-2 blocks as our primary anesthetic technique. 

Regional anesthesia has the advantages of immediate perioperative pain control with reduced opioid consumption, reduced use of muscle relaxants, reduced sympathetic activation, and enhanced postoperative recovery with earlier mobilization and discharge [11]. While there are many positive outcomes, risks include local anesthetic toxicity and neural injury [12]. Block failure may be more common in less experienced anesthesiologists and therefore having dedicated experienced block teams improves efficacy. 

The nature and location of surgery, patients’ medical comorbidities, as well as the surgeon, dictated the type of block that can be performed. In many cases, a neuraxial technique such as spinal or epidural anesthesia can be implemented. However, in our case series, the neuraxial technique was contraindicated for both patients due to the tendency for systemic hypotension, as well as the risk of an epidural hematoma or abscess [13]. Another available option was the transversus abdominis plane (TAP) block. Previous studies describe that QL blocks offer increased dermatomal coverage than TAP blocks, and more reliable coverage of the iliohypogastric, ilioinguinal, and genitofemoral femoral nerves [5]. The key differences between the two are depth and location of injection. A posterior TAP block is superficial to the TAP and its aponeurosis while a QL occurs deep to the transversus abdominis aponeurosis [14]. Different types of QL blocks lead to slightly different levels of anesthesia as previously described. This is secondary to the location of the local anesthetic infiltration (anterolateral border of the QL at the junction of the QL with the transversalis fascia for a QL1 and into the LIFT at the posterior aspect of the QL muscle for a posterior QL block), the posterior spread of this block is thought to have increased dermatomal coverage with spread of the local anesthetic to the paravertebral space for visceral analgesia [14]. For these reasons we elected to perform a posterior QL block.

Posterior QL block was selected for surgical anesthesia in this case in order to provide adequate coverage of both somatic and visceral structures. A TAP block would not have provided adequate surgical anesthesia as it only reliably covers the somatic innervation of the lateral flank [15]. Rectus sheath block would again have only covered the somatic innervation of the midline of the abdomen. Thoracic paravertebral blocks would have provided the necessary somatic and visceral analgesia, but were avoided as the patient was coagulopathic. Erector spinae plane block may have provided sufficient anesthesia for this procedure, however, there have been no reports of this in the literature and so was not utilized for this case. However, there is evidence to show that posterior QL blocks do provide visceral and somatic anesthesia, and so were selected for use in this case [16]. QL type 3, or anterior QL block, is known to have a more frequent incidence of quadriceps weakness than posterior QL block with similar anesthetic coverage, and so was not considered for this procedure [17-18].

Our case series is one of few in which QL blocks have been utilized as the primary anesthetic. For the posterior QL block to be successful it is essential that anesthesia staff are trained and comfortable with this technique. The patient’s pathology and proposed surgical intervention should also be amenable to posterior QL blockade. We describe the successful utilization of the posterior QL blocks in two high risk multi-morbid patients who, without this block would have been unsuitable for urgent hernia repair. Morbidity and mortality were zero and effective post-operative pain control was achieved. We demonstrate that regional anesthesia, in particular posterior QL blocks, can be utilized in the patient population where underlying comorbid conditions make general or neuraxial anesthesia prohibitively high risk. Future studies are recommended and should focus on determining the efficacy and safety of posterior QL blocks for open hernia repairs in a wider high-risk patient population.

## Conclusions

Patients who are unable to be medically optimized for a general anesthetic prior to emergent and/or urgent surgical procedures should be offered other forms of anesthesia. Neuraxial anesthesia may also be contraindicated as in this case series. Therefore, regional anesthetics such as QL blocks can be safely utilized as a primary anesthetic in this patient population.
